# Preliminary assessment of serum capillary zone electrophoresis in the Asian elephant (*Elephas maximus*)

**DOI:** 10.3389/fvets.2023.1204880

**Published:** 2023-09-07

**Authors:** Shirley Osorio, Jeny Soto, Dennis Schmitt, Wendy Kiso, Carolyn Cray

**Affiliations:** ^1^Division of Comparative Pathology, Department of Pathology and Laboratory Medicine, University of Miami Miller School of Medicine, Miami, FL, United States; ^2^White Oak Conservation, Yulee, FL, United States; ^3^Missouri State University, Springfield, MO, United States

**Keywords:** serum protein electrophoresis, capillary zone electrophoresis, albumin, acute phase protein, method comparison, reference intervals, Asian elephant, *Elephas maximus*

## Abstract

Serum protein electrophoresis has been demonstrated to have utility in diagnostic workup, wellness exams, and prognosis. Agarose gel electrophoresis (AGE) has previously been described for use with serum from Asian elephants (*Elephas maximus*). As the newer method of capillary zone electrophoresis (CZE) is becoming more commonplace in veterinary diagnostic laboratories, serum samples from Asian elephants were examined using this method. CZE allowed for a reproducible definition of two beta fractions and, overall, showed a low coefficient of variation for fraction quantitation. Preliminary reference intervals were generated using samples primarily from an older population of 22 female elephants. Albumin levels determined by CZE were also compared with those determined by the bromocresol green method on a chemistry analyzer. It was found that the latter method overestimated the level of albumin with a mean positive bias of 11.6% or 0.38 g/dL, thus method-specific reference intervals should be used. Significant negative correlations were observed between A/G ratio determined by CZE and serum amyloid A levels (r = −0.47, *p* < 0.0001) and haptoglobin (*r* = −0.52, *p* < 0.0001); both APP were significantly correlated with the alpha 2 globulin fraction (p < 0.0001). CZE reflects an overall picture of changes in acute phase proteins and immunoglobulins and accurate quantitation of albumin and thus should be considered as an adjunct tool to the use of other measures of the acute phase response in patient monitoring.

## Introduction

Serum protein electrophoresis (SPEP) of non-domesticated mammals can provide a broad picture in a health assessment including the detection of inflammation and polyclonal gammopathies and the accurate quantitation of albumin (1, 2). In the Asian elephant (*Elephas maximus*), SPEP has previously been described using modern agarose gel electrophoresis (AGE) methods, and older reports have also included electrophoresis data in studies and health assessments of this species and the African elephant (*Loxodonta africana*) (3–6). Importantly, the IUCN lists this species as endangered, and many challenges have been identified in the preservation of this species in the wild and under managed care (7, 8). Validated and impactful clinical pathology methods are important to aid in this mission.

Reference intervals have been published for the Asian elephant with a notation that the methods and implementation have been variable (3). Recently, capillary zone electrophoresis (CZE) has become more available in reference laboratories, and veterinary applications have begun to be investigated (1). This method provides increased ease of use with full automation and utilizes an ultraviolet detector for the quantitation of fractions; this results in higher resolution and reduced coefficient of variation (9). Mammals studied by this method to date include mouse, rat, dog, cat, marmoset, panda, and dolphin (10–13).

Other testing methods are available to quantitate acute phase proteins (APPs) including albumin, an important negative acute phase protein, and positive acute phase proteins such as serum amyloid A (SAA) and haptoglobin (HP). The latter has been previously detailed in the Asian elephant (3, 14). The primary chemistry analyzer method for albumin quantitation involves the use of bromocresol green (BCG) dye. This method has previously been found to result in inaccurate results in non-traditional species, and electrophoresis has been proposed to be the gold standard method (1).

The goals of the current study were as follows: (1) to define the CZE-derived serum protein electrophoretogram of the Asian elephant, (2) to generate preliminary reference intervals, and (3) to evaluate the correlation of the data with other methods of albumin and acute phase protein quantitation.

## Method

### Animals

Samples were obtained from one facility in 2020–2021 and included 30 Asian elephants with a median age of 37 years (95% CI: 23–47). There were 27 female and 3 male elephants. Of this group, 22 elephants were classified as clinically healthy based on physical exam and bloodwork including hematology and biochemistry. This group, used for the generation of preliminary reference intervals, remained healthy for a minimum of 3 months after samples were acquired and were all female with a median age of 32 years (95% CI, 22–45). A total of 166 samples were examined for the portion of the study on the albumin method and SAA/HP comparisons. This included all elephants and repeated measures, regardless of clinical status. Clinical abnormalities and diagnoses included retained placenta, abscess, trauma, hematuria, edema, anorexia, constipation, foot infection, abdominal pain, arthritis, tuberculosis, and recto-vaginal fistula.

### Samples

Samples were obtained from Asian elephants undergoing health examinations using standard phlebotomy techniques, where 6–9 mL of venous blood was collected from the ear vein and placed into red top vacutainer tube and allowed to clot. The serum was separated by centrifugation (10 min; 2,479 x g) and shipped overnight in the cold pack to the University of Miami (Miami, FL) for analysis.

### Sample analysis

Capillary zone electrophoresis was performed by the Capillarys 2 Flex Piercing System according to the manufacturer’s protocols (Sebia, Norcross, GA United States). In brief, samples were diluted 1:8 using urine running buffer as diluent. Absolute values for each fraction were obtained by multiplying the percentages for each fraction by the total protein concentration as determined using the biuret method and a Vitros 5600 chemistry analyzer (Ortho Vitros Diagnostics, Rochester, NY, United States). The A/G ratio was calculated by dividing the sum of prealbumin and albumin by the sum of alpha, beta, and gamma globulins. The coefficient of variation (CV) for fraction quantitation ranged from 1.1 to 2.4% for albumin, alpha 2, beta, and gamma globulins and 6.0–6.7% for prealbumin and alpha 1 globulin. The bromocresol green method for albumin determination was also performed by the Vitros 5600 analyzer. The CV was 1%. SAA and HP were analyzed as previously described on the Vitros 5600 analyzer (3). All analyzers were maintained according to the manufacturer’s guidelines, and quality control samples were run before test sample analysis.

### Statistical analysis

Reference intervals (RIs) were determined following the guidelines set forth by The American Society for Veterinary Clinical Pathology (ASVCP) quality assurance and laboratory standards committee guidelines for the determination of reference intervals in veterinary species (15). RIs were calculated using MedCalc version 20.022 (Ostend, Belgium). Outliers, determined by Tukey’s analysis, were not removed.

Other statistical analyses were conducted using MedCalc and GraphPad Prism 6.07 (GraphPad Software, San Diego, CA 92108). For the comparison of methods, BCG, SAA, and HP data were non-Gaussian in distribution using the D’Agostino–Pearson test, so Spearman’s correlation and Wilcoxon matched-pairs signed rank test were used. *p*-values of <0.05 were considered significant. CV analysis was performed using eight replicate tests of a single sample performed within 1 day.

## Results

**Figure 1:**
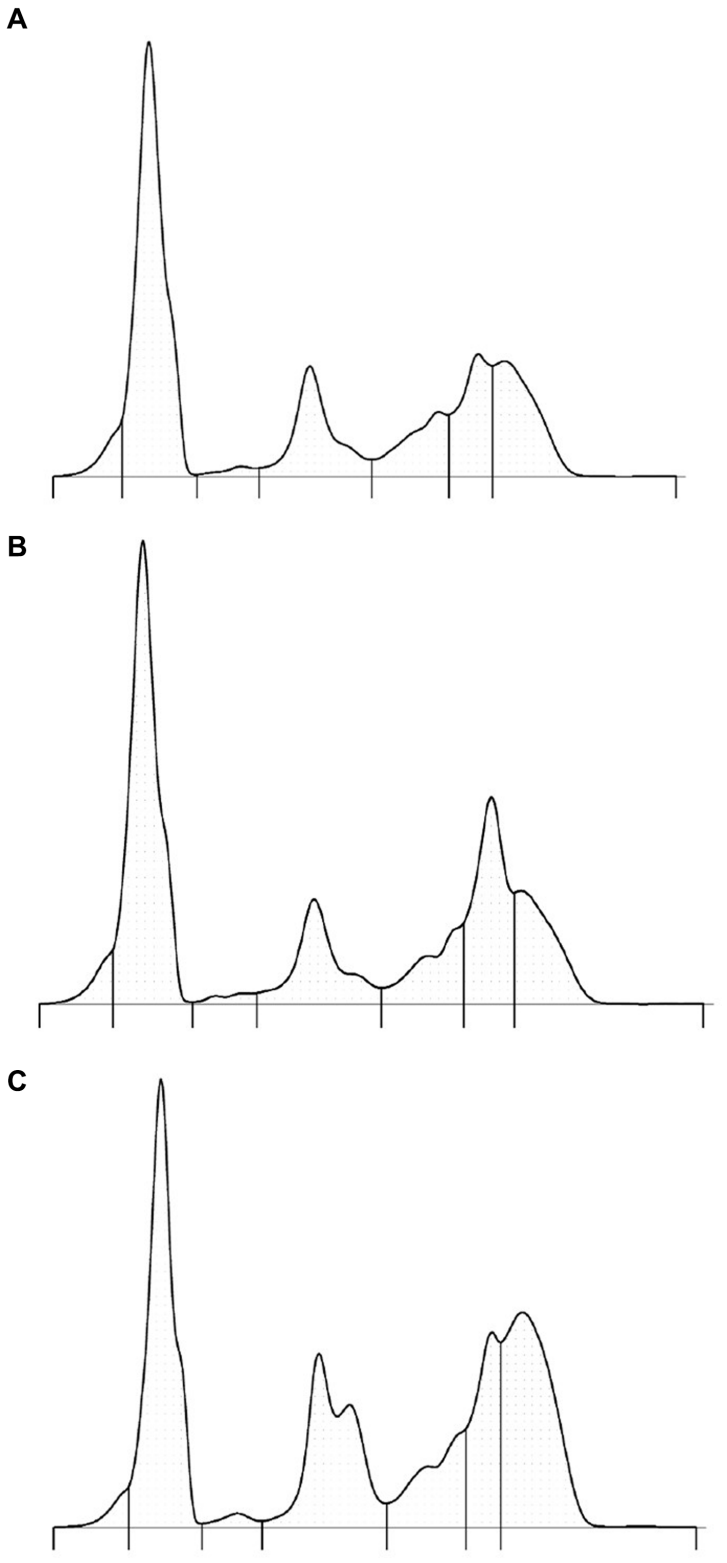
Protein electrophoretograms from a representative clinically normal elephant—serum sample **(A)**, paired plasma sample **(B)**, and serum from an elephant with a partially retained placenta **(C)**. The respective A/G ratios are as follows: 0.84, 0.75, and 0.45. The SAA level in sample C was 153 mg/L (normal <42.5 mg/L).

The use of capillary zone electrophoresis resolved a minimum of seven fractions including two beta globulin fractions in clinically normal Asian elephants (Figure 1). The beta 2 fraction was increased in plasma versus serum samples (Figures 1A,B). In an Asian elephant with a retained partial placenta (Figure 1C), increases in alpha 2, beta, and gamma globulins were present. Reference intervals were calculated using data obtained from 22 individual clinically normal animals (Table 1).

**Table 1:** Reference intervals for capillary zone electrophoresis measurands and total protein for the Asian elephant (*n* = 22).

Measurand	Mean	SD	Median	Min	Max	Normality, value of *p*	RI	CI 90% LRL	CI 90% URL
Total protein, g/dL	8.2	0.4	8.2	7.7	9.2	NG, 0.04	7.3–9.0	7.0–7.6	8.6–9.3
A/G ratio	0.69	0.10	0.67	0.55	0.90	G, 0.42	0.47–0.89	0.41–0.52	0.81–0.96
Prealbumin, g/dL	0.21	0.07	0.19	0.11	0.38	NG, 0.04	0.03–0.33	0–0.09	0.28–0.39
Prealbumin, %	2.5	0.7	2.3	1.4	4.1	G, 0.22	0.7–3.9	0.4–1.3	3.3–4.5
Albumin, g/dL	3.12	0.24	3.12	2.66	3.59	G, 0.90	2.59–3.62	2.47–2.74	3.46–3.78
Albumin, %	38.1	3.6	38.2	31.7	45.2	G, 0.65	30.2–45.8	28.3–32.4	43.3–47.9
Alpha 1, g/dL	0.09	0.02	0.09	0.06	0.11	G, 0.70	0.06–0.12	0.05–0.07	0.11–0.13
Alpha 1, %	1.1	0.2	1.1	0.7	1.4	G, 0.62	0.7–1.5	0.6–0.8	1.4–1.6
Alpha 2, g/dL	1.11	0.09	1.11	0.92	1.25	G, 0.86	0.92–1.30	0.87–0.98	1.24–1.35
Alpha 2, %	13.7	1.2	13.4	11.5	15.6	G, 0.88	11.1–16.0	10.4–11.8	15.2–16.7
Beta 1, g/dL	0.86	0.19	0.86	0.57	1.24	G, 0.55	0.44–1.26	0.34–0.55	1.13–1.39
Beta 1, %	10.5	2.2	10.5	7.2	15.7	G, 0.44	5.6–15.0	4.3–6.9	13.4–16.5
Beta 2, g/dL	1.18	0.17	1.19	0.85	1.44	G, 0.32	0.83–1.54	0.72–0.94	1.44–1.64
Beta 2, %	14.4	1.8	14.5	11.1	17.1	G, 0.15	10.5–18.4	9.5–11.6	17.3–19.6
Beta total, g/dL	2.04	0.30	1.97	1.63	2.62	G, 0.35	1.31–2.63	1.21–1.51	2.40–2.88
Beta total, %	24.9	3.2	24.1	20.6	32.6	G, 0.20	16.9–31.1	15.4–19.0	28.4–34.1
Gamma, g/dL	1.64	0.34	1.64	1.16	2.60	NG, 0.03	0.87–2.30	0.66–1.14	2.06–2.57
Gamma, %	19.9	3.4	20.2	14.7	28.6	G, 0.39	12.2–27.0	10.3–14.7	24.6–29.2

No outliers were removed. The robust method was used to generate the intervals.

The A/G ratio calculated from CZE was compared with SAA and HP levels. The Spearman r for SAA was −0.47 (*p* < 0.0001) and for HP was −0.52 (*p* < 0.0001). When the APP was compared with the individual globulin fractions, both SAA and HP had the highest significant positive correlation with the alpha 2 globulin fraction. The Spearman r for SAA was 0.54 (*p* < 0.0001) and for HP was 0.57 (*p* < 0.0001).

**Figure 2:**
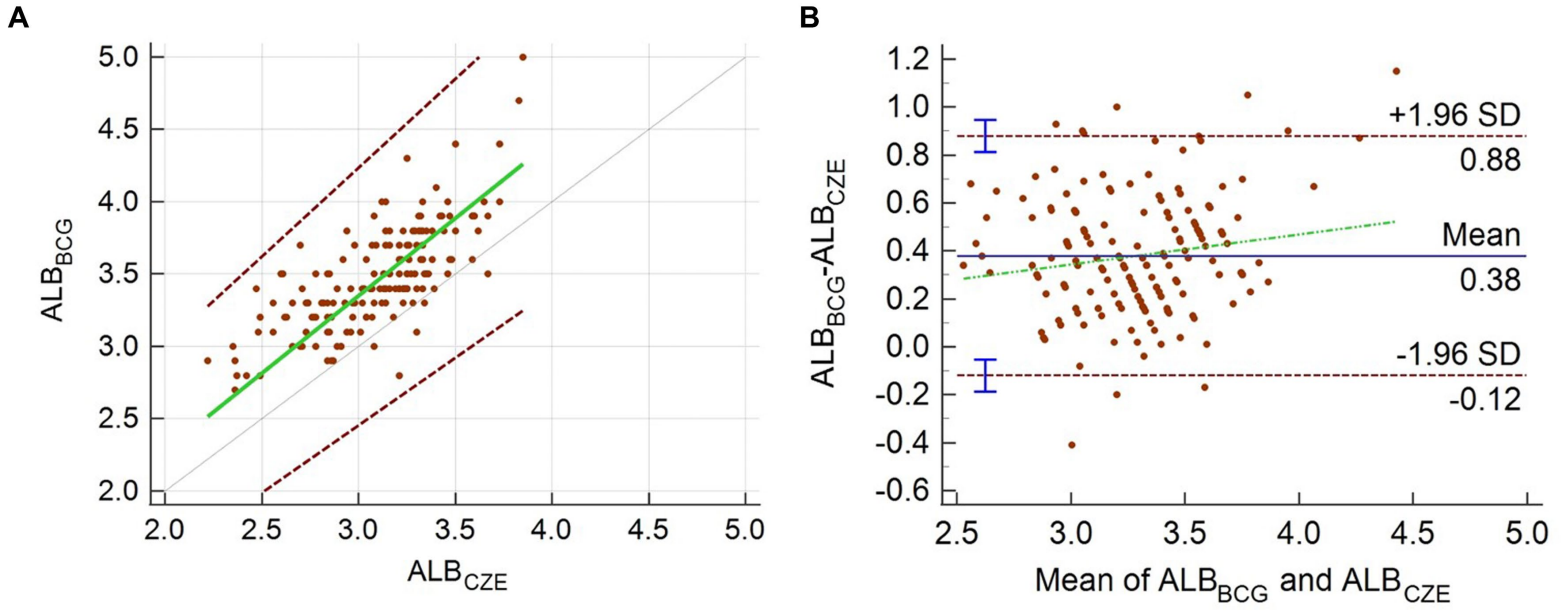
Passing–Bablok regression analysis **(A)** and Bland–Altman plot **(B)** for the comparison of albumin measured by the CZE and BCG methods. In panel **(A)**, the green line is the regression line, and the dotted gray line is the line of identity; the two dashed lines show the 95% limits of agreement. In panel **(B)**, the mean percentage and limits of agreement are shown. The green dashed line is the regression line of the differences between the methods.

The difference between albumin measured by BCG assay and that derived from CZE is shown in Figure 2. Albumin measured by the BCG assay ranged from 2.7 to 5.0 g/dL with a mean of 3.5 g/dL and median of 3.4 g/dL. Albumin measured by CZE ranged from 2.2 to 3.9 g/dL with a mean and median of 3.1 g/dL. The data from the BCG method were significantly correlated with that from CZE (*r* = 0.73, *p* < 0.0001), and the BCG method produced significantly higher results versus CZE (*p* < 0.0001). The difference between these measures is shown in Figure 2. The equation of the regression line (with 95% CI in parentheses) was y = 0.136 (−0.346–0.551) + 1.071x (0.933–1.23). No proportional or constant error was detected as the 95% confidence intervals for the slope and y-intercept included 1.0 and 0.0, respectively. However, a mean positive bias (for the BCG method) of 0.38 g/dL was evident by the Bland–Altman plot.

## Discussion

Serum protein electrophoresis of Asian elephants was previously reported using modern AGE methods (3). By both AGE and CZE methods, the normal electrophoretogram of this species is characterized by a dominant albumin band and bridged beta and gamma globulin fractions. In CZE, the improved resolution of protein fractions including identification of new fractions and easier definition of existing fractions has previously been reported in human and animal species (9–12, 16–18). In the current study, two beta globulin fractions were consistently present, and a prominent alpha 2 globulin fraction by CZE was observed (3). The location of the beta fraction was aided by the comparison of paired serum and plasma samples (Figures 1A,B). Two subfractions of alpha 2 globulins could be routinely observed by the presence of an acute phase response (Figure 1C). Notably, the coefficient of variation using CZE was very low (<2.5%) for fractions that composed more of the electrophoretogram; the less well-defined prealbumin and alpha 1 globulin fractions varied by 6%. The prealbumin fraction was consistently observed as a shoulder to the albumin fraction but, in some elephants, was clearly defined. It is unknown if this fraction represents true prealbumin or other acute phase proteins; however, given its mobility anodic to albumin, the term prealbumin is considered appropriate (1). Overall, the presence and appearance of this fraction is variable in mammals including panda, dolphin, mouse, rhinoceros, and dog, among others, where CZE methods have been described (10–12, 18). The alpha 1 globulin fraction was often a less well-defined area in clinically normal elephants versus a defined band in clinically abnormal elephants (Figure 1C). A slight shoulder migrating cathodic to albumin was consistently observed. This subfraction has been observed in other species and has been proposed in birds to be apolipoprotein A1 (10, 19). Overall, based on fraction migration and fraction delimit placements, the alpha globulin fractions appeared especially different from the AGE method (3). It is important to emphasize that the electrophoresis method-specific reference intervals should be used in conjunction with consistent placement of fraction delimits (1).

As protein electrophoresis can reflect an ongoing acute phase response, additional statistical examination was performed using data obtained for SAA and HP. These two APPs were previously defined as major and minor APPs, respectively, in the Asian elephant (3, 14). Both markers were negatively correlated with the A/G ratio, and while the correlation was significant, it was only moderate. This finding was expected as these methods are not equivalent and are reflective of the influence of more than 200 acute phase proteins including albumin and immunoglobulins on the A/G ratio (1). While marked increases in SAA levels can occur with inflammation, it is not expected that these changes would be clearly visible in the electrophoretogram. However, it is interesting that both SAA and HP were significantly correlated with the alpha 2 globulin fraction. Although HP is believed to migrate in the alpha fraction in other mammals, this finding and that of SAA have not been described for the elephant (2). Additional proteomic studies and analysis of more electrophoretograms from clinically abnormal elephants may better address this observation. Overall, protein electrophoresis and acute phase protein quantitation should be recognized as two different tools in patient monitoring and assessment.

Protein electrophoresis has been recognized as the gold standard method for the quantitation of albumin, as the use of the BCG method in chemistry analyzers has previously been shown to be problematic due to the reaction of the dye with globulins (1, 20). While the impact of globulins can be significant in non-mammalian species, this method has also been previously demonstrated to be influenced by variable binding affinity to albumin (21). In the Asian elephant, the mean positive bias of the BCG method compared with CZE of 11.6% or 0.38 g/dL. This bias is lower than the recommended total allowable error of 15% (22). However, it does support the previously acknowledged bias of the BCG method and reiterates the need for method-specific reference intervals (20, 21, 23).

The current study has been limited by the small sample size of mostly older female Asian elephants from a single herd. Future studies may address differences by species and age. In addition, the examination of samples from animals with differing clinical abnormalities and repeated measures in tandem with acute phase proteins and other biomarkers will aid in judging the overall clinical utility of this method in elephants under managed care.

## Data availability statement

The original contributions presented in the study are included in the article/supplementary material, further inquiries can be directed to the corresponding author.

## Ethics statement

All testing was performed using leftover samples from planned health assessments. The study plan was reviewed and approved by the facility which contributed the samples (White Oak Conservation). The study was conducted in accordance with the local legislation and institutional requirements.

## Author contributions

SO, JS, DS, WK, and CC performed the conceptualization. SO and JS carried out laboratory processing and evaluation. DS and WK provided and analyzed supporting animal information. CC analyzed the data statistically. CC wrote the initial version of the manuscript. SO, JS, DS, WK, and CC edited the manuscript. All authors contributed to the article and approved the submitted version.

## Funding

This study was supported internally through funds from the Division of Comparative Pathology.

## Conflict of interest

The authors declare that the research was conducted in the absence of any commercial or financial relationships that could be construed as a potential conflict of interest.

## Publisher’s note

All claims expressed in this article are solely those of the authors and do not necessarily represent those of their affiliated organizations, or those of the publisher, the editors and the reviewers. Any product that may be evaluated in this article, or claim that may be made by its manufacturer, is not guaranteed or endorsed by the publisher.
